# Prenatal and childhood exposure to phthalate diesters and sex steroid hormones in 2-, 5-, 8-, and 11-year-old children: A pilot study of the Taiwan Maternal and Infant Cohort Study

**DOI:** 10.1016/j.je.2016.10.009

**Published:** 2017-05-30

**Authors:** Hui-Ju Wen, Lillian Sie, Pen-Hua Su, Chia-Jui Chuang, Hsiao-Yen Chen, Chien-Wen Sun, Li-Hua Huang, Chao Agnes Hsiung, Shu-Li Julie Wang

**Affiliations:** a National Institute of Environmental Health Sciences, National Health Research Institutes, Miaoli, Taiwan; b Department of Pediatrics, Chung Shan Medical University Hospital, Taichung, Taiwan; c Manufacturing Technology Center, Taiwan Semiconductor Manufacturing Company, Hsinchu, Taiwan; d Department of Nursing, Chung Shan Medical University Hospital, Taichung, Taiwan; e Institute of Population Health Sciences, National Health Research Institutes, Miaoli, Taiwan; f Department of Public Health, College of Public Health, China Medical University, Taichung, Taiwan; g Research Center for Environmental Medicine, Kaohsiung Medical University, Kaohsiung, Taiwan; h Department of Public Health, National Defense Medical Center, Taipei, Taiwan

**Keywords:** Phthalate diester, Sex steroid hormone, Birth cohort, Children, Endocrine disruptor

## Abstract

**Background:**

Phthalate diesters are commonly used and have been well established as environmental endocrine disruptors. However, few studies have examined their effects on sex steroid hormones in children. We followed children over time to examine the association between pre- and post-natal phthalate exposure and sex steroid hormone levels at 2, 5, 8, and 11 years of age.

**Methods:**

We recruited 430 pregnant women from central Taiwan from 2000 to 2001 and assessed their children at birth, 2, 5, 8, and 11 years of age. We studies children with at least one measurement for both phthalate and hormone levels during each any of the follow-up time point (n = 193). Estradiol, free testosterone, testosterone, and progesterone were measured from venous blood. Three monoesters of di-2-ethylhexyl phthalate (DEHP), mono-benzyl phthalate, mono-n-butyl phthalate, mono-ethyl phthalate, and mono-methyl phthalate were measured in maternal urine collected during the 3rd trimester and child urine collected at each follow-up point. The sum of mono-2-ethylhexyl phthalate (∑MEHP) was calculated by summing the concentrations of the three DEHP monoesters. Generalized estimating equation regression analysis with repeated measures was used to estimate associations between phthalate metabolites and hormone levels.

**Results:**

After adjustment for potential confounders, maternal ∑MEHP level was associated with decreased levels of progesterone in girls (β = −0.309 p = 0.001). The child ∑MEHP concentration was associated with decreased levels of progesterone for girls (β = −0.194, p = 0.003) and with decreased levels of free testosterone for boys (β = −0.124, p = 0.004).

**Conclusions:**

Early-life DEHP exposure may alter sex steroid hormones of children over time, which may pose potential reproductive health risks.

## Highlights

• Prenatal phthalate exposure was associated with decreased PG levels in girls.
• Postnatal DEHP exposure was related to decreased free TT in boys and PG in girls.
• Use of phthalate-containing products in developing children should be limited.

## Introduction

Phthalate esters are a class of chemicals added to an extensive range of products, including plastics and lotions.^1^, ^2^ Owing to their ubiquity, people are constantly exposed to phthalate esters through ingestion, inhalation, and dermal contact; however, the effects of phthalates on human reproductive health remain unclear.

The balance of sex steroid hormone levels in the somatic nervous system is regulated and controlled by the hypothalamus-pituitary-gonadal (HPG) axis, a neuroendocrine axis that includes the hypothalamus, the anterior pituitary gland, and the gonads. In general, gonadotropin releasing hormone (GnRH) neurons in the hypothalamus induce the secretion of GnRH; GnRH then stimulates the anterior pituitary to synthesize and release luteinizing hormone (LH) and follicle stimulating hormone (FSH) to the gonads. The gonads (i.e., ovaries in females and testes in males) subsequently synthesize and release sex steroid hormones, mainly estradiol and progesterone in females and testosterone in males, to the somatic circulation. The HPG axis is controlled through a negative feedback mechanism: systemic sex steroid hormone concentrations inhibit pituitary responsiveness to GnRH and GnRH secretion in the hypothalamus. Exposure to phthalate diester, an established endocrine-disrupting chemical, may interfere with normal functioning of the HPG axis and cause reproductive dysfunction.^3^, ^4^, ^5^, ^6^, ^7^

Phthalate diester is reported to have anti-androgenic and weak estrogenic effects.^8^, ^9^, ^10^, ^11^, ^12^, ^13^ Small children may be particularly prone to exposure because of frequent hand-to-mouth activity and increased phthalate exposure dose per kilogram of body weight due to small body size. Swan and colleagues found that boys born to mothers with increased urinary levels of phthalate metabolites had reduced anogenital distance.^14^ Maternal urinary phthalate metabolite levels during pregnancy were found to be associated with decreased sex steroid levels in newborns.^15^ Di-2-ethylhexyl phthalate (DEHP) exposure is also linked to gynecomastia in boys and earlier age at pubarche for boys^8^ and for girls.^16^, ^17^ A previous study of older children showed that di-n-butyl phthalate (DnBP) is negatively associated with adrenal androgen levels in boys.^16^ For girls, increased urinary phthalate levels are associated with delayed pubarche^9^, ^13^, ^18^; however, evidence on the effects of phthalates on thelarche is less conclusive.^19^, ^20^, ^21^

The objective of this prospective birth cohort study was to examine the association between maternal urinary phthalate metabolite levels during pregnancy (prenatal exposure) and childhood sex steroid hormone levels.

## Materials and methods

### Study participants

**Fig. 1:**
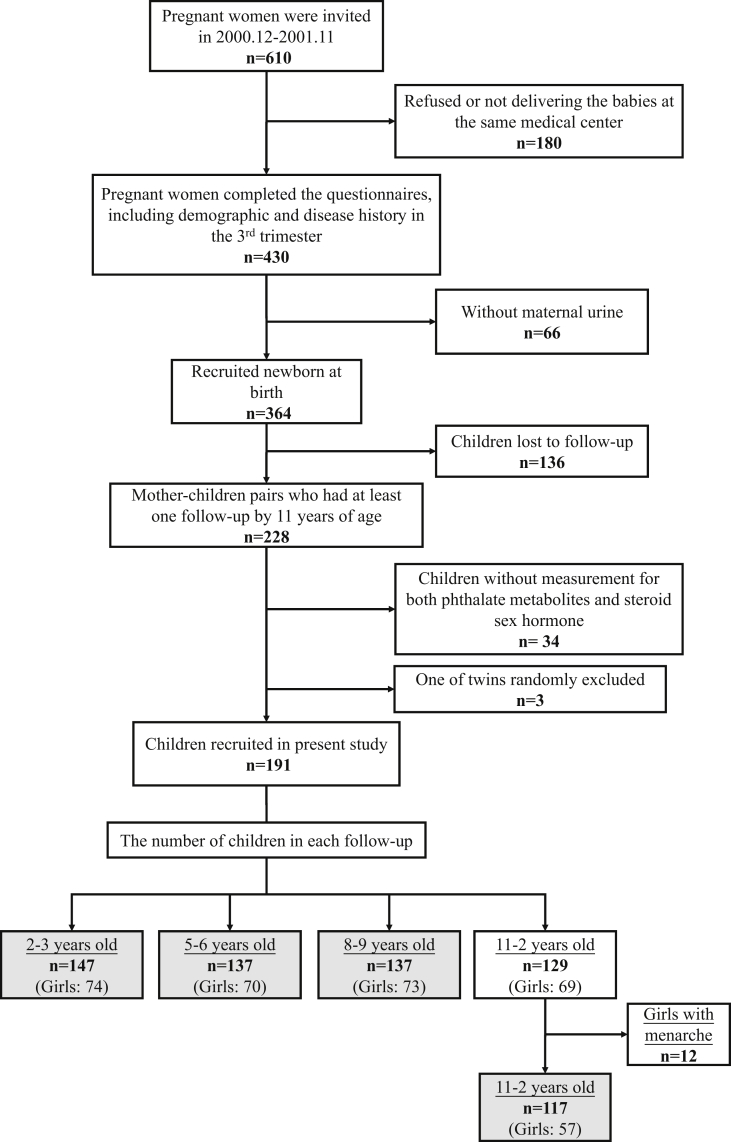
Flow chart of participant recruitment.

Pregnant women between the ages of 25 and 35 years without clinical complications who were part of the pilot study of the Taiwan Maternal Infant Cohort Study (TMICS) were recruited for this study. A total of 610 women in their third trimester of pregnancy in a regional hospital in central Taiwan were invited to join the study, and 430 women (75%) agreed to be interviewed (Fig. 1). Interviews were performed after subjects gave informed consent to participate in the study. A total of 364 newborns whose mothers had provided a maternal urine sample in the 3rd trimester were recruited in the follow-up study (Fig. 1). Children were assessed when they were 2–3 (in 2003), 5–6 (in 2006), 8–9 (in 2009), and 11–12 (in 2012) years of age. Written consent was obtained from the children, in addition to the main caretaker, when they were 6 years of age or older at the time of follow-up. The study process was approved by the Research Ethics Committee of the National Health Research Institutes and Chung Shan Medical University Hospital in Taiwan.

### Data collection

All pregnant women completed a questionnaire that included questions on maternal age, parity, education level, disease history, and dietary and smoking habits. Maternal urine was collected from subjects during the third trimester of pregnancy (28–38 weeks). Blood and urine were collected from the children at each follow-up visit. Urine collection methods used for the children are detailed in a previous publication by Lin et al.^22^ Urine samples of mothers and children were collected and stored in brown glass bottles. We also did the blank test to check for phthalate contamination.

### Measurement of phthalate metabolites and sex steroid hormones

Urine concentrations (μg/L) of seven metabolites of the five most commonly used phthalate esters (mono-2-ethylhexyl phthalate [MEHP], mono-2-ethyl-5-hydroxyhexyl phthalate [MEHHP], and mono-2-ethyl-5-oxohexyl phthalate [MEOHP] for DEHP, mono-benzyl phthalate [MBzP] for benzyl butyl phthlate [BBzP], mono-n-butyl phthalate [MnBP] for DnBP, mono-ethyl phthalate [MEP] for diethyl phthalate [DEP]), and mono-methyl phthalate [MMP] for dimethyl phthlate [DMP]) were analyzed with quantitative liquid chromatography-tandem mass spectrometry (LC-MS/MS), as described in a previous study.^15^, ^23^ Briefly, we prepared 0.1 mL urine sample aliquots containing 1 M ammonium acetate (20 μL), β-glucuronidase (10 μL), and a mixture of isotopic phthalate metabolite standards. The samples were incubated at 37 °C for 1.5 h. Each sample was injected with 270 μL solvent (0.1% formic acid and 5% acetonitrile) in glass screw-cap vials and mixed for quantitative LC-MS/MS after hydrolysis.^23^

The sum of the MEHP levels (∑MEHP) was estimated as the sum of MEHP, MEHHP, and MEOHP. Urinary creatinine levels were measured at Kaohsiung Medical University Chung-Ho Memorial Hospital using a spectrophotometric method.^22^ Phthalate metabolite measurements were divided by urinary creatinine levels and expressed as “μg/g creatinine” to account for urinary volume correction.

Estradiol (pg/mL), testosterone (ng/mL), free testosterone (pg/mL), and progesterone (ng/mL) in venous blood were measured using radioimmunoassays (Diagnostic Products Corporation, Los Angeles, CA, USA). Due to the limited quantity of blood collected from 2- and 3-year-old children, data on progesterone were not available for this group.

Phthalate metabolite levels and sex steroid hormone concentrations under the detection limits were conventionally assigned a value of half the limit of detection (LOD) value. The LOD value of phthalate metabolites and sex steroid hormones were 0.55, 0.23, 0.26, 0.99, 1.6, 3.4, and 2.2 ng/mL for MEHP, MEHHP, MEOHP, MBzP, MnBP, MMP, and MEP and 1.5 ng/mL, 0.15 pg/mL, 2.2 pg/mL, and 0.1 ng/mL for testosterone, free testosterone, estradiol, and progesterone, respectively. The percentage of above the LOD value on phthalates metabolites and sex steroid hormones in children is reported in eTable 1.

### Statistical analysis

Statistical analyses were conducted using SPSS software version 20 (IBM, Armonk, NY, USA) and JMP software version 10.0 (SAS Institute Inc., Cary, NC, USA). Influential outlier points were excluded from the analysis (eTable 2) based on sensitivity analyses. Geometric means and percentiles of metabolites and hormones were calculated. Wilcoxon rank-sum test was used to test for the differences in metabolite and hormone levels between sexes.

Values for all metabolites were natural log-transformed due to skewness in variable distributions and high standard errors. Values of testosterone, free testosterone, and estradiol levels were also natural log-transformed to achieve normal distributions needed for generalized estimating equation (GEE) linear regression analysis.

To estimate the overall associations of prenatal and childhood phthalate exposure with hormone levels in children at ages 2–3, 5–6, 8–9, and 11–12 years, a GEE linear regression analysis with repeated measures using an unstructured correlation matrix was conducted with outliers excluded in the sensitivity analysis. The GEE model was adjusted for prenatal and childhood phthalate exposure as the primary exposures of interest. Potential confounders were included in the model if inclusion changed the main coefficient estimates by 10% or more. Because multiple comparisons were done to examine the relationships between phthalate exposure and sex steroid hormones, the α value was adjusted, and a *P* value ≤0.0083 (i.e., 0.05 divided by 6) was considered statistical significant.

## Results

A total of 191 children who had at least one follow-up at 2–3, 5–6, 8–9, and 11–12 years, and had measurements for both phthalate metabolites and hormone levels were included in the final analysis. For mothers with multiple newborns, data for only one child was randomly selected for analysis (Fig. 1). We further excluded girls (n = 12) who had menarche at the age of 11–12 years. The characteristics of included children and their mothers are reported in Table 1. The maternal characteristics of age, BMI, weight gain during pregnancy, education level, environmental tobacco smoke (ETS) exposure, and alcohol drinking habits did not differ between boys and girls. Compared to newborn girls, newborn boys had greater mean weight (mean 3194.0 [standard deviation {SD}, 379.4] g versus 3022.7 [SD, 465.2] g) and length (mean 51.78 [SD, 2.13] cm versus 50.77 [SD, 2.68] cm) upon delivery. Individuals who refused to participate did not differ in maternal age, smoking habit, ETS exposure status, children's sex, birth order, birth outcomes, and maternal urinary phthalate metabolite concentrations from those who enrolled. However, those successfully followed had higher maternal education and weight gain during pregnancy (eTable 3).

**Table 1:** Characteristics of mothers and their newborns by children sex (n = 191).

	Female (n = 97)		Male (n = 94)		*P* value^a^
	n	Mean (SD) or n (%)	n	Mean (SD) or n (%)	
Mothers					
Age, years^b^	92	29.11 (4.64)	87	29.08 (3.42)	0.822
Pre-pregnancy BMI, kg/m^2^^b^	92	20.97 (3.02)	86	20.29 (2.70)	0.202
Gestational weight gain^b^	92	11.11 (5.42)	82	12.17 (5.13)	0.149
Education					
<12 years	92	37 (40.2)	87	34 (36.2)	0.917
≥12 years		55 (59.8)		53 (56.4)	
Smoking during pregnancy					
Yes	92	2 (2.1)	87	0	–
No		90 (92.8)		87 (92.6)	
ETS exposure before pregnancy					
Yes	91	43 (44.3)	87	39 (41.5)	0.746
No		48 (49.5)		48 (51.1)	
Alcohol drinking in pregnancy					
Yes	92	3 (3.1)	87	4 (4.3)	0.715
No		89 (91.8)		83 (88.3)	
Newborns					
Gestational week, weeks^b^	72	38.54 (1.88)	65	38.91 (1.27)	0.300
Birth weight, g^b^	73	3022.7 (465.2)	67	3194.0 (379.4)	0.013
Birth length, cm^b^	72	50.77 (2.68)	67	51.78 (2.13)	0.030
Birth head circumference, cm^b^	71	33.33 (1.46)	67	33.64 (1.15)	0.224
Birth order					
1st	73	38 (39.2)	81	50 (53.2)	0.656
2nd		26 (26.8)		23 (24.5)	
≥3rd		9 (9.3)		8 (8.5)	
Method of delivery					
Vaginal birth	73	26 (26.8)	67	27 (28.7)	0.846
Vacuum delivery		24 (24.7)		20 (21.3)	
Cesarean section		23 (23.7)		20 (21.3)	

BMI, body mass index; ETS, environmental tobacco smoke; SD, standard deviation.

Some numbers do not add up to the total n because of missing values.

“–”: not applicable for analysis.

a *P* value obtained from Wilcoxon rank-sum test, Chi-square test, or Fisher's exact test to test for gender differences.

b Mean (SD).

Descriptive statistics for phthalate metabolites and sex steroid hormones levels stratified by age and sex are reported in Table 2. Aside from MMP and MBzP, the geometric mean for urinary phthalate levels decreased with increasing child age. Moreover, the geometric mean for sex steroid hormone concentrations increased with increasing child age in general. Although testosterone levels in 5-year-olds were higher than those in 8-year-olds, the difference was not statistically significant and might be caused by chance. Sex differences were statistically significant in testosterone and estradiol levels for measurements at 8 and 11 years old, in free testosterone levels for measurement at 5 and 11 years old, and in progesterone levels for measurement at 5 and 8 years old. We originally assessed correlations between sex steroid hormones and phthalate levels stratified by age group. The correlation pattern was the same across all age groups; therefore, the summarized GEE results were reported.

**Table 2:** Descriptive statistics of phthalates metabolite levels (μg/g creatinine), urinary creatinine (mg/dL), and sex steroid hormones stratified by age and sex.

	Girls					Boys					*P* value^a^
	N	Geometric mean	25th	50th	75th	N	Geometric mean	25th	50th	75th	
*Phthalates metabolites, μg/g creatinine*^b^											
∑MEHP^c^											
Maternal^d^	97	48.33	26.17	44.63	85.58	93	58.04	28.26	56.56	100.80	0.246
2 years	53	167.68	93.16	142.70	289.70	61	173.27	107.20	175.50	276.60	0.621
5 years	61	145.35	81.46	123.60	253.00	61	139.82	84.88	117.60	223.90	0.906
8 years	68	86.99	50.12	67.59	124.30	59	95.92	52.90	86.60	154.80	0.473
11 years	53	65.10	43.62	62.66	91.49	54	70.88	45.24	69.31	102.40	0.511
MEHP											
Maternal^d^	97	17.45	8.12	16.12	33.28	93	18.90	9.71	18.72	33.65	0.403
2 years	53	15.47	9.22	14.27	30.90	61	14.86	8.88	15.66	23.55	0.937
5 years	61	11.80	5.34	12.42	24.63	61	10.55	6.81	10.89	16.88	0.546
8 years	68	7.74	3.06	5.72	15.81	59	9.19	3.98	9.07	17.86	0.235
11 years	53	7.80	5.18	9.01	19.19	54	9.67	5.21	10.50	17.97	0.527
MBzP											
Maternal^d^	97	17.34	9.46	18.29	28.92	93	15.29	10.07	16.50	25.58	0.496
2 years	53	6.75	3.65	7.53	11.66	61	6.79	3.02	8.28	15.02	0.842
5 years	61	12.64	6.77	11.26	21.63	61	16.34	10.27	14.80	29.75	0.054
8 years	68	10.14	5.36	7.74	17.93	59	11.64	4.89	10.85	24.33	0.386
11 years	53	2.99	1.63	2.92	6.34	54	3.51	2.02	3.50	7.08	0.385
MnBP											
Maternal^d^	97	73.68	35.51	60.09	167.20	93	72.33	37.79	69.08	131.90	0.941
2 years	53	163.35	100.20	169.10	256.50	61	162.56	93.53	179.10	261.50	0.755
5 years	61	98.27	69.76	102.50	139.40	60	127.26	64.99	98.03	160.30	0.750
8 years	68	102.09	65.89	90.83	150.50	59	83.51	47.59	77.04	148.00	0.155
11 years	53	48.04	35.07	48.88	76.90	54	52.54	40.28	53.47	70.28	0.586
MEP											
Maternal^d^	97	66.65	35.67	62.88	109.40	93	61.15	34.19	57.67	103.30	0.570
2 years	53	35.91	17.73	32.75	63.13	61	26.05	13.55	24.13	54.23	0.138
5 years	61	17.84	10.03	15.19	35.50	61	21.73	10.90	17.65	40.03	0.429
8 years	68	18.12	8.62	17.76	27.07	59	14.27	8.26	11.05	23.88	0.140
11 years	53	6.75	0.66	8.76	37.69	54	9.65	4.25	12.25	22.17	0.594
MMP											
Maternal^d^	97	52.11	26.92	56.44	99.77	93	49.01	29.26	51.39	92.16	0.696
2 years	53	14.34	8.67	15.72	23.47	61	14.76	8.80	16.50	23.59	0.825
5 years	61	15.33	8.91	12.77	27.70	60	16.40	7.60	16.32	33.97	0.610
8 years	68	7.57	4.01	7.33	14.02	59	6.47	3.90	5.89	10.92	0.282
11 years	53	11.25	5.36	13.04	24.17	54	10.42	5.00	14.75	28.35	0.978
*Creatinine, mg/dL*											
Maternal^d^	97	54.48	30.82	59.17	94.53	93	58.73	33.14	61.33	98.34	0.754
2 years	53	33.54	21.58	31.78	62.88	61	42.50	27.97	52.19	74.49	0.071
5 years	61	45.77	25.45	60.90	90.60	61	50.96	30.15	48.90	93.65	0.925
8 years	68	28.36	19.70	28.80	46.55	59	49.39	29.80	56.70	85.70	<0.0001
11 years	53	53.20	30.85	50.50	90.70	54	73.71	44.95	95.30	121.40	0.016
*Sex steroid hormones*^b^											
Testosterone, ng/mL											
2 years	46	3.17	2.58	3.15	4.13	47	3.29	2.60	3.20	4.30	0.729
5 years	64	6.43	5.44	6.35	7.47	60	5.93	5.08	5.72	7.09	0.053
8 years	63	3.71	2.87	3.87	4.80	56	3.23	2.55	3.10	4.25	0.026
11 years	52	6.10	4.19	6.22	8.19	53	14.18	3.78	10.02	52.16	0.046
Free testosterone, pg/mL											
2 years	46	0.16	0.13	0.17	0.20	48	0.16	0.12	0.16	0.22	0.671
5 years	64	0.20	0.14	0.18	0.24	60	0.15	0.12	0.14	0.20	0.006
8 years	65	0.25	0.21	0.24	0.30	57	0.24	0.21	0.24	0.28	0.568
11 years	51	0.22	0.17	0.22	0.32	53	0.42	0.17	0.34	0.98	0.006
Estradiol, pg/mL											
2 years	46	8.31	7.48	8.40	9.40	46	7.82	7.08	7.65	8.82	0.077
5 years	63	7.09	5.49	7.91	9.42	60	6.59	5.07	6.93	9.03	0.265
8 years	63	11.08	9.71	11.40	12.58	55	10.40	9.01	10.09	11.57	0.036
11 years	52	15.93	12.08	15.04	21.94	53	10.70	9.10	10.65	12.51	<0.0001
Progesterone, ng/mL											
5 years	17	0.24	0.15	0.23	0.37	14	0.15	0.11	0.15	0.20	0.018
8 years	69	0.31	0.19	0.35	0.51	62	0.25	0.16	0.26	0.36	0.031
11 years	51	0.43	0.32	0.43	0.54	53	0.39	0.27	0.40	0.53	0.304

MBzP, mono-benzyl phthalate; MEHP, mono-2-ethylhexyl phthalate; MEHHP, mono-2-ethyl-5-hydroxyhexyl phthalate; MEOHP, mono-2-ethyl-5-oxohexyl phthalate; MEP, mono-ethyl phthalate; MMP mono-methyl phthalate; MnBP, mono-n-butyl phthalate.

Some numbers do not add up to total n because of missing values.

a *P* value obtained from Wilcoxon rank-sum test to for the mean difference between boys and girls.

b All concentrations of phthalate metabolites, creatinine, and sex steroid hormones were natural log-transformed.

c ∑MEHP = MEHP + MEHHP + MEOHP.

d Measured from urine collected during the third trimester of pregnancy.

The beta statistics from the GEE analyses for sex steroid hormone levels in relation to maternal urinary phthalate metabolite levels are reported in Table 3. Among girls, maternal ∑MEHP, MEHP, MBzP, and MEP exposures were negatively associated with progesterone levels (β = −0.309 [standard error {SE}, 0.092], p = 0.001 for ∑MEHP; β = −0.205 [SE, 0.050], p < 0.001 for MEHP; β = −0.275 [SE, 0.064], p < 0.001 for MBzP, and β = −0.269 [SE, 0.101], p = 0.008 for MEP). Among boys, no significant association was found between maternal phthalate exposure and sex steroid hormone, indicating that maternal urinary phthalate levels were associated with decreased sex steroid hormone levels in girls but not in boys.

**Table 3:** Betas from the generalized equation estimate (GEE) linear regression for sex steroid hormone levels in relation to maternal urinary phthalate metabolite levels (μg/g creatinine).^a^^,^^b^

*Hormone*^c^	Girls		Boys	
Phthalate^c^	β (SE)	*P* value^d^	β (SE)	*P* value^d^
*Testosterone, ng/mL*				
∑MEHP	−0.044 (0.075)	0.555	0.087 (0.050)	0.084
MEHP	0.068 (0.124)	0.584	0.037 (0.050)	0.466
MBzP	0.089 (0.115)	0.438	1.316 (0.587)	0.025
MnBP	0.178 (0.163)	0.274	0.079 (0.081)	0.326
MEP	0.048 (0.050)	0.334	0.027 (0.061)	0.663
MMP	−0.008 (0.078)	0.916	−0.026 (0.064)	0.681
*Free Testosterone, pg/mL*				
∑MEHP	−0.019 (0.054)	0.731	0.058 (0.101)	0.562
MEHP	0.050 (0.078)	0.515	−0.010 (0.048)	0.833
MBzP	0.079 (0.110)	0.473	0.461 (0.275)	0.094
MnBP	0.048 (0.081)	0.553	0.073 (0.077)	0.345
MEP	0.018 (0.056)	0.744	−0.029 (0.059)	0.625
MMP	0.011 (0.062)	0.855	−0.047 (0.059)	0.419
*Estradiol (pg/mL)*				
∑MEHP	−0.086 (0.057)	0.13	−0.014 (0.057)	0.801
MEHP	−0.193 (0.156)	0.215	−0.018 (0.022)	0.422
MBzP	−0.100 (0.142)	0.482	0.007 (0.032)	0.821
MnBP	−0.032 (0.028)	0.247	−0.010 (0.023)	0.675
MEP	−0.012 (0.059)	0.839	−0.022 (0.022)	0.322
MMP	−0.166 (0.100)	0.097	0.001 (0.023)	0.981
*Progesterone (ng/mL)*				
∑MEHP	−**0.309 (0.092)**	**0.001**	−0.030 (0.082)	0.72
MEHP	−**0.205 (0.050)**	**<0.0001**	−0.015 (0.040)	0.70
MBzP	−**0.275 (0.064)**	**<0.0001**	0.033 (0.064)	0.608
MnBP	−0.232 (0.122)	0.057	0.072 (0.051)	0.162
MEP	−**0.269 (0.101)**	**0.008**	0.019 (0.045)	0.672
MMP	−0.205 (0.118)	0.081	0.010 (0.039)	0.803

BMI, body mass index; MBzP, mono-benzyl phthalate; MEHHP, mono-2-ethyl-5-hydroxyhexyl phthalate; MEHP, mono-2-ethylhexyl phthalate; MEOHP, mono-2-ethyl-5-oxohexyl phthalate; MEP, mono-ethyl phthalate; MnBP, mono-n-butyl phthalate; MMP mono-methyl phthalate; SE, standard error.

∑MEHP = MEHP + MEHHP + MEOHP.

a Maternal urinary metabolite concentration during the third trimester of pregnancy.

b Model was adjusted for child's levels of phthalate metabolites, age and BMI at time of follow-up, and maternal age, education, BMI, smoking and drinking habits during third trimester of pregnancy.

c Natural log-transformed in model.

d p < 0.0083 indicates a statistical significant association and is shown in bold.

The beta statistics generated from the GEE analyses for sex steroid hormone levels in relation to children urinary metabolite levels are reported in Table 4. Testosterone levels were inversely associated with MnBP among girls (β = −0.364 [SE, 0.084], p < 0.0001) and MBzP among boys (β = −0.382 [SE, 0.135], p = 0.005). Moreover, MMP was negatively associated free testosterone levels in girls (β = −0.091 [SE, 0.033], p = 0.006). ∑MEHP was negatively associated free testosterone levels in boys (β = −0.124 [SE, 0.044], p = 0.004).

**Table 4:** Betas from the generalized equation estimate (GEE) linear regression for sex steroid hormone levels in relation to children urinary phthalate metabolite levels (μg/g creatinine).^a^

*Hormone*^b^	Girls		Boys	
Phthalate^b^	β (SE)	*P* value^c^	β (SE)	*P* value^c^
*Testosterone, ng/mL*				
∑MEHP	−0.054 (0.063)	0.396	−0.088 (0.063)	0.161
MEHP	−0.095 (0.052)	0.069	0.018 (0.055)	0.742
MBzP	0.038 (0.046)	0.407	**−0.382 (0.135)**	**0.005**
MnBP	**−0.364 (0.084)**	**<0.0001**	−0.014 (0.066)	0.834
MEP	0.013 (0.058)	0.824	0.029 (0.071)	0.685
MMP	−0.102 (0.080)	0.200	0.049 (0.054)	0.366
*Free Testosterone, pg/mL*				
∑MEHP	−0.105 (0.059)	0.075	**−0.124 (0.044)**	**0.004**
MEHP	0.034 (0.049)	0.485	0.009 (0.046)	0.845
MBzP	0.094 (0.043)	0.030	0.022 (0.099)	0.826
MnBP	−0.178 (0.153)	0.243	−0.025 (0.045)	0.577
MEP	0.064 (0.028)	0.024	0.006 (0.049)	0.903
MMP	**−0.091 (0.033)**	**0.006**	−0.058 (0.048)	0.224
*Estradiol, pg/mL*				
∑MEHP	**−0.115 (0.036)**	**0.002**	−0.017 (0.028)	0.549
MEHP	−0.082 (0.061)	0.175	−0.052 (0.023)	0.021
MBzP	0.140 (0.077)	0.071	**−0.056 (0.017)**	**0.001**
MnBP	−0.035 (0.041)	0.389	−0.034 (0.028)	0.218
MEP	**0.165 (0.058)**	**0.004**	0.008 (0.017)	0.641
MMP	−0.067 (0.089)	0.456	−0.007 (0.017)	0.691
*Progesterone, ng/mL*				
∑MEHP	**−0.194 (0.066)**	**0.003**	0.002 (0.058)	0.973
MEHP	0.004 (0.090)	0.967	0.017 (0.039)	0.658
MBzP	0.016 (0.092)	0.860	−0.041 (0.040)	0.303
MnBP	0.003 (0.252)	0.991	−0.053 (0.059)	0.361
MEP	−0.016 (0.052)	0.763	−0.001 (0.028)	0.971
MMP	0.022 (0.129)	0.863	−0.063 (0.032)	0.046

BMI, body mass index; MBzP, mono-benzyl phthalate; MEHHP, mono-2-ethyl-5-hydroxyhexyl phthalate; MEHP, mono-2-ethylhexyl phthalate; MEOHP, mono-2-ethyl-5-oxohexyl phthalate; MEP, mono-ethyl phthalate; MMP mono-methyl phthalate; MnBP, mono-n-butyl phthalate; SE, standard error.

∑MEHP = MEHP + MEHHP + MEOHP.

a Model was adjusted for child's age and BMI at time of follow-up, and maternal levels of phthalate metabolites, age, education, BMI, smoking and drinking habits during third trimester of pregnancy.

b Natural log-transformed in model.

c p < 0.0083 indicates a statistical significant association and is shown in bold.

Estradiol levels among girls were inversely associated ∑MEHP (β = −0.115 [SE, 0.036], p = 0.002) (Table 4). Among boys, MBzP was associated with decreasing estradiol levels (β = −0.056 [SE, 0.017], p = 0.001). In addition, progesterone levels among girls was negatively associated with ∑MEHP (β = −0.194 [SE, 0.066], p = 0.003). Overall, childhood phthalate exposure (excluding MEP) was associated with decreased testosterone, free testosterone, estradiol, and progesterone.

## Discussion

In the present study, we used an 11-year birth cohort to determine the association between prenatal and childhood phthalate exposure and children sex steroid hormone levels. We found that prenatal DEHP, DBzP, and DEP exposures were associated with decreased levels of progesterone in girls. Childhood DEHP, DBzP, DnBP, and DMP exposure was associated with decreased levels of testosterone, free testosterone, estradiol, and progesterone. The anti-androgenic effects of phthalate exposure in the present results confirms findings from previous animal and epidemiological studies.^8^, ^10^, ^13^, ^14^, ^24^, ^25^

To our knowledge, this is the first completed longitudinal study that detected an association between prenatal and postnatal phthalate exposure and sex steroid hormones from infancy to early adolescence, with adjustment for childhood phthalate exposure. A study of adolescent girls in Australia examining the effects of prenatal phthalate exposure on reproductive hormones found an anti-androgenic effect.^26^ However, exposure was estimated in serum and without data on childhood exposure.

In the present study, maternal ∑MEHP, MEHP, MBzP, and MEP exposures were inversely associated with progesterone levels in girls. The results were consistent with those of Araki et al, who reported that DEHP exposure in utero was inversely associated with progesterone levels in children from the Hokkaido birth cohort study in Japan.^27^ These findings are in line with animals studies in which rat granulosa cells treated with DEHP had inhibited progesterone synthesis.^28^ In our previous studies, we found that maternal urinary levels of DEHP were linked with reduced uterus size in girls aged 8 and 11 years old.^29^ The effects of prenatal exposure persisted years after delivery, adding to the growing body of evidence indicating that exposure may have consequences on subsequent sexual development in children,^29^ which could have an impact on reproductive health. Other studies have shown evidence of impaired reproductive development related to changes in hormone levels.^9^, ^13^, ^18^, ^30^ Given the broad exposure of phthalate esters and the vulnerability of developing children, these findings are worth noting. Changes in phthalate use during pregnancy could be warranted if the future reproductive health of children is at risk.

In the present study, childhood levels of ∑MEHP and MBzP were inversely associated with free testosterone and testosterone levels in boys, respectively. These findings are consistent with the anti-androgenic effects of phthalates documented in experimental animal and observational human studies.^10^, ^12^, ^31^, ^32^, ^33^ DEHP and DBP have been linked with abnormal testicular development, lowered sperm counts, and decreased testosterone levels in male rats due to their toxicity to leydig cells, the site of androgen synthesis^10^, ^31^; similar results have been detected in adult men.^12^, ^32^ Pan et al found that concentrations of urinary phthalate metabolites were negatively associated with levels of testosterone, free testosterone, and LH in 1066 Chinese adult men.^32^ Anti-androgenic and anti-estrogenic effects of exposure were also detected in girls in our study. Childhood levels of MnBP and MMP were associated with decreased testosterone and free testosterone levels in girls, respectively. These findings are similar to those of studies that revealed decreased adrenal androgen levels and delayed pubarche in girls with high phthalate exposure.^16^ Given the limited number of studies providing evidence of anti-androgenic effects in girls,^9^, ^16^, ^18^, ^19^, ^26^ further studies should be conducted to enhance understanding of these effects.

Childhood levels of ∑MEHP were inversely associated with estradiol levels in girls. Our findings are also in line with those of a study that found an inverse association between DEHP and age of menarche among Australian adolescent girls^26^ and studies that found delayed thelarche in girls with higher phthalate metabolite levels.^19^, ^21^ An anti-estrogenic effect of phthalate exposure was also detected in boys exposed to MBzP, which was inversely associated with estradiol levels. Our results support the hypothesis that phthalate exposure is anti-estrogenic. Previous studies conducted in female rats found both mildly estrogenic and anti-estrogenic effects from exposure to various phthalates.^11^, ^34^ Since testosterone is a precursor to estradiol, the anti-androgenic effects of phthalate exposure may play a role in lowering estradiol secretion levels.^28^

In this study, the geometric means of all metabolites except MEP and MBzP in children at each follow-up point were higher than what was reported in the NHANES survey of children 6–11 years old in the United States.^25^ Median levels of MEHP in our study population at different age were higher than those of German nursery school children aged 2–6 years old. However, MnBP and MBzP levels were lower.^22^, ^35^ The extensive use of plastic products in Taiwan may explain the relatively higher levels of metabolites in this study population.^36^ With the exception of MMP and MBzP, metabolite levels decreased with increasing age. This trend is consistent with that in other populations, particularly when urinary phthalate levels were creatinine-corrected.^37^, ^38^

There were some limitations of this study. Due to the short half-life of phthalates, the urinary measurements may not accurately reflect actual exposure over time. We have found correlations between phthalate levels across different age groups. We found some consistency with exposure over time by testing correlations between the phthalate metabolites. Misclassification may have occurred when estimating prenatal exposure, since maternal urine was only collected in the third trimester; however, another study found that urinary levels of MBP, MEP, MBzP, and MEHP were consistent throughout pregnancy.^39^ Since the potential misclassification of exposure is non-differential across all age and sex groups, the effects detected in this study may be underestimated because the bias would be towards the null hypothesis. Another limitation was the small quantity of blood drawn from children 2–3 years old; progesterone levels could not be analyzed due to a lack of adequate sample volume. Finally, approximately 55.6% of mother–infant pairs were excluded from the final analysis due to mothers without urine samples, children loss to follow-up, and children without data of both phthalate metabolites and sex steroid hormones. Selection bias might be a concern. However, the characteristics of the infants (e.g., gender, birth outcomes, or birth order) and mothers (e.g., maternal age, smoking habit, or ETS exposure status) and the concentrations of maternal urinary phthalate metabolites did not differ significantly between children that were followed up and those were not, except maternal education and weight gain during pregnancy (eTable 3). Moreover, since the interviewers and the main caretakers were unaware of the main research hypothesis, selection bias caused by differential participation was less likely.

Despite these limitations, the study has several strengths. Because of its longitudinal design with multiple measurements of exposure and outcome, we were able to observe consistent associations between phthalate exposure and altered sex steroid hormone concentrations in children at different ages. We also adjusted the regression models for confounding factors of childhood phthalate exposure when estimating the association between prenatal exposure and sex steroid hormone levels.

In conclusion, we found that childhood phthalate exposure was associated with decreased levels of testosterone, estradiol, and progesterone. Prenatal phthalate exposure was associated with decreased levels of sex steroid hormones in girls. We suggest that the use of phthalate products during pregnancy and childhood may alter sex steroid hormone levels in growing children. Determining whether or not such effects are associated with later reproductive function in adolescents requires further assessment.

## Conflicts of interest

None declared.
