# Supplementary material for: Prenatal and childhood exposure to phthalate diesters and sex steroid hormones in 2-, 5-, 8-, and 11-year-old children: A pilot study of the Taiwan Maternal and Infant Cohort Study
Source: J Epidemiol. 2017 May 30;27(11):516–23. doi: 10.1016/j.je.2016.10.009 (PMC5608604; doi:10.1016/j.je.2016.10.009)
Supplement: Supplementary file 1 [file mmc1.pdf]

eTable 1. The percentage of above the limit of detection value on phthalates metabolite levels and sex steroid hormones stratified by age and sex

	LOD	Girls		Boys	
		n	% >LOD	n	% >LOD
Phthalate metabolites, ng/mL					
MEHP					
Maternal[a]	0.55	97	100.0	93	100.0
2 years	0.7	53	98.1	61	100.0
5 years	0.7	61	90.2	61	98.4
8 years	0.7	68	97.1	59	100.0
11 years	0.7	53	86.8	54	96.3
MBzP					
Maternal[a]	0.99	97	100.0	93	97.8
2 years	0.3	53	90.6	61	90.2
5 years	0.3	61	100.0	61	100.0
8 years	0.3	68	95.6	59	100.0
11 years	0.3	53	83.0	54	92.6
MnBP					
Maternal[a]	1.66	97	100.0	93	100.0
2 years	1	53	100.0	61	100.0
5 years	1	61	100.0	60	100.0
8 years	1	68	100.0	59	100.0
11 years	1	53	66.0	54	85.2
MEP					
Maternal[a]	2.15	97	100.0	93	100.0
2 years	0.3	53	100.0	61	96.7
5 years	0.3	61	100.0	61	100.0
8 years	0.3	68	100.0	59	100.0
11 years	0.3	53	100.0	54	100.0
MMP					
Maternal[a]	3.42	97	93.8	93	92.5

2 years	0.3	53	96.2	61	98.4
5 years	0.3	61	100.0	60	98.3
8 years	0.3	68	98.5	59	100.0
11 years	0.3	53	94.3	54	90.7
Sex steroid hormone					
Testosterone, ng/mL					
2 years	0.15	46	100.0	47	100.0
5 years	0.15	64	100.0	60	100.0
8 years	0.15	63	100.0	56	100.0
11 years	0.15	52	100.0	53	100.0
Free testosterone, pg/mL					
2 years	0.22	46	84.8	48	83.3
5 years	0.22	64	89.1	60	78.3
8 years	0.22	65	98.5	57	98.2
11 years	0.22	51	86.3	53	94.3
Estradiol, pg/mL					
2 years	1.5	46	100.0	46	100.0
5 years	1.5	63	100.0	60	100.0
8 years	1.5	63	100.0	55	100.0
11 years	1.5	52	100.0	53	100.0
Progesterone, ng/mL					
5 years	0.1	17	100.0	14	100.0
8 years	0.1	69	100.0	62	100.0
11 years	0.1	51	100.0	53	100.0

LOD, limit of detection; MBzP, mono-benzyl phthalate; MEHHP, mono-2-ethyl-5-hydroxyhexyl phthalate; MEHP, mono-2-ethylhexyl phthalate; MEOHP, mono-2-ethyl-5-oxohexyl phthalate; MEP, mono-ethyl phthalate; MMP mono-methyl phthalate; MnBP, mono-n-butyl phthalate.

[a] Measured from urine collected during the third trimester of pregnancy

eTable 2. Outlier points identified by outlier influential point sensitivity analysis: excluded data

Age	Gender	Hormone	Metabolite	Value
8	Male	Estradiol	-	87.4 pg/mL
5	Female	Estradiol	-	88.6 pg/mL
5	Female	Progesterone	-	7.34 ng/mL
8	Female	-	MBzP	1594.1 µg/creatinine
5	Male	-	MnBP	16,929.9 µg/creatinine
5	Male	-	MMP	3381.9 µg/creatinine

eTable 3. Characteristics of mothers and children in included pairs (n=191) and excluded pairs (n=239)

Characteristic	Included pairs		Excluded pairs		p-value[a]
	n	mean (SD) or n (%)	n	mean (SD) or n (%)	
**Mothers**					
Age, years[b]	185	29.08 (4.03)	233	28.30 (4.40)	0.1507
Pre-pregnancy BMI, kg/m^2[b]	189	20.67 (2.88)	228	20.64 (3.25)	0.604
Gestational weight gain[b]	186	11.80 (5.36)	228	13.14 (6.40)	0.041
Education					
<12 years	191	77 (40.3)	236	128 (53.6)	0.004
≥12 years		114 (59.7)		108 (45.2)	
Smoking during pregnancy					
Yes	191	2 (1.0)	236	6 (2.5)	0.306
No		189 (99.0)		230 (96.2)	
ETS exposure before pregnancy					
Yes	190	85 (44.5)	234	114 (47.7)	0.414
No		105 (55.0)		120 (50.2)	
Alcohol drinking in pregnancy					
Yes	191	8 (4.2)	236	6 (2.5)	0.344
No		183 (95.8)		230 (96.2)	
**Newborns**					
Sex					
Girl	191	97 (50.8)	151	71 (29.7)	0.489
Boy		94 (49.2)		80 (33.5)	
Gestational age, weeks[b]	144	33.67 (1.62)	129	38.67 (1.44)	0.437
Birth weight, g[b]	147	3097 (432.4)	130	3083 (472.9)	0.857
Birth length, cm[b]	146	51.19 (2.46)	130	51.17 (2.53)	0.987
Birth head circumference, cm[b]	145	33.48 (1.34)	129	33.37 (1.57)	0.282
Birth order					
1st	178	97 (50.8)	53	30 (12.6)	0.933

2nd		62 (32.5)		17 (7.1)	
≥3rd		19 (9.9)		6 (2.5)	
Method of delivery					
Vaginal birth	147	55 (28.8)	130	33 (13.8)	0.081
Vacuum delivery		45 (23.6)		52 (21.8)	
Cesarean section		47 (24.6)		45 (18.8)	
**Maternal urinary phthalate metabolites (μg/mL)[c]**					
∑MEHP[d]	191	50.33 (80.33)	177	44.99 (49.92)	0.928
MEHP	191	12.46 (13.59)	177	13.55 (14.44)	0.943
MBzP	191	11.94 (10.67)	177	12.68 (12.62)	0.100
MnBP	191	66.39 (77.95)	177	61.60 (66.54)	0.779
MEP	191	56.03 (69.79)	177	47.52 (48.09)	0.838
MMP	191	48.19 (55.02)	177	52.71 (47.73)	0.235

BMI, body mass index; ETS, environmental tobacco smoke; MBzP, mono-benzyl phthalate; MEHHP, mono-2-ethyl-5-hydroxyhexyl phthalate; MEHP, mono-2-ethylhexyl phthalate; MEOHP, mono-2-ethyl-5-oxohexyl phthalate; MEP, mono-ethyl phthalate; MMP mono-methyl phthalate; MnBP, mono-n-butyl phthalate; SD, standard deviation.

[a] P-value of different between included and excluded pairs was calculated by Wilcoxon rank-sum test for continue variables and χ^2 test or Fisher's exact test for categorical variables

[b] mean (SD)

[c] Measured from urine collected during the third trimester of pregnancy

[d] ∑MEHP = MEHP + MEHHP + MEOHP

Some numbers do not add up to total n because of missing values.
